# Studies on phytochemical, antioxidant, anti-inflammatory, hypoglycaemic and antiproliferative activities of *Echinacea purpurea* and *Echinacea angustifolia* extracts

**DOI:** 10.1080/13880209.2016.1265989

**Published:** 2016-12-13

**Authors:** Rayn Clarenc Aarland, Angel Ernesto Bañuelos-Hernández, Mabel Fragoso-Serrano, Edgar del Carmen Sierra-Palacios, Fernando Díaz de León-Sánchez, Laura Josefina Pérez-Flores, Fernando Rivera-Cabrera, José Alberto Mendoza-Espinoza

**Affiliations:** a Postgraduate Program in Experimental Biology, Universidad Autónoma Metropolitana, Iztapalapa, Ciudad de México, México;; b Departamento de Ciencias de la Salud DCBS, Universidad Autónoma Metropolitana-Iztapalapa, Ciudad de México, México;; c Departamento de Farmacia, Facultad de Química, Universidad Nacional Autónoma de México, Ciudad de México, México;; d Colegio de Ciencias y Humanidades, Universidad Autónoma de la Ciudad de México, Ciudad de México, México

**Keywords:** Herbal, standardization, fingerprints

## Abstract

**Context:***Echinacea* (Asteraceae) is used because of its pharmacological properties. However, there are few studies that integrate phytochemical analyses with pharmacological effects.

**Objective:** Evaluate the chemical profile and biological activity of hydroalcoholic *Echinacea* extracts.

**Materials and methods:** Density, dry matter, phenols (Folin–Ciocalteu method), flavonoids (AlCl_3_ method), alkylamides (GC-MS analysis), antioxidant capacity (DPPH and ABTS methods), antiproliferative effect (SRB assay), anti-inflammatory effect (paw oedema assay, 11 days/Wistar rats; 0.4 mL/kg) and hypoglycaemic effect (33 days/Wistar rats; 0.4 mL/kg) were determined in three *Echinacea* extracts which were labelled as A, B and C (A, roots of *Echinacea purpurea* L. Moench; B, roots, leaves, flowers and seeds of *Echinacea purpurea*; C, aerial parts and roots of *Echinacea purpurea* and roots of *Echinacea angustifolia* DC).

**Results:** Extract C showed higher density (0.97 g/mL), dry matter (0.23 g/mL), phenols (137.5 ± 2.3 mEAG/mL), flavonoids (0.62 ± 0.02 mEQ/mL), and caffeic acid (0.048 mg/L) compared to A and B. A, B presented 11 alkylamides, whereas C presented those 11 and three more. B decreased the oedema (40%) on day 2 similar to indomethacin. A and C showed hypoglycaemic activity similar to glibenclamide. Antiproliferative effect was only detected for C (IC_50_ 270 μg/mL; 8171 μg/mL; 9338 μg/mL in HeLa, MCF-7, HCT-15, respectively).

**Discussion and conclusion:** The difference in the chemical and pharmacological properties among extracts highlights the need to consider strategies and policies for standardization of commercial herbal extracts in order to guarantee the safety and identity of this type of products.

## Introduction

In order to meet their basic needs, man has always maintained a close relationship with plants. This relationship resulted in the emergence of indigenous and traditional medicine (Koehn & Carter 2005; Jones et al. 2006). Traditional medicine continues to play an important role in continents such as Africa, Asia and America. The World Health Organization (WHO) has paid attention to the success achieved by Eastern countries such as China, where medicinal plants were incorporated into the official medicine, resulting in their clinical evaluation (Aarland et al. 2014). In this regard, in recent years the Food and Drug Administration (FDA) has approved three treatments based on herbal mixtures that includes antiallergenic, anticancer and antipsoriatic drugs (Newman & Cragg 2012, 2016).

However, even with this background, one of the main problems that hinder the inclusion of medicinal plants in the National and International Pharmacopoeias, is the lack of studies that validate their use through the chemical and pharmacological description of the mixtures used in the final extracts (Pokorny & Schmidt 2001; Nindo et al. 2003; Hossain et al. 2010; Gupta et al. 2011). Our research group proposes that an important step in order to develop phytopharmaceutical products from medicinal plants is the standardization of the extracts. This standardization process is important since it allows having a product with the same physical and chemical characteristics, assuring the same pharmacological effect. Table 1 shows the proposed method, the same that was taken as the basis for the development of this study (Aarland et al. 2014).

**Table 1.** Study design.

1 Physical	
a) Dry matter	
b) Density	
2 Chemical	
a) Qualitative analyses	*Saponins*
	*Coumarines*
	*Anthraquinones*
	*Tannins*
	*Alkaloids*
b) Quantitative analyses	*Total phenols*
	*Total flavonoids*
	*Caffeic acid*
	*Chlorogenic acid*
	*Alkylamides*
3 Biological	
a) *In vitro* anti-oxidant capability	*DPPH*
	*ABTS^*+*^*
b) Cytotoxicity	*MCF-7, HeLa, HCT-15*
c) *In vivo* hypoglycemiant effect	*Male Wistar rats*
d) *In vivo* anti-inflammatory effect	*Male Wistar rats*

The first reports of the use of the plants belonging to the genus *Echinacea* (Asteraceae) date to the beginning of the twentieth century, currently the sales associated with the hydroalcoholic extracts of *Echinacea purpurea* (L.) Moench, *Echinacea pallida* (Nutt.) Nutt. and *Echinacea angustifolia* DC. reach 21 million dollars in the USA (Blumenthal et al. 2005). Sales data in combination with its pharmacological properties make *Echinacea* an interesting study model to apply the methodology of standardization described earlier in Table 1.

Ethnobotanical studies made by WHO refer among the documented empirical uses of *Echinacea*, its use against snakebites, to heal wounds and as a primitive antibiotic (World Health Organization 1999). In pharmacological studies validated experimentally and published in specialized literature, there are reports of antiviral, anticancer activity, and immunomodulatory effects (Chicca et al. 2007; Pellati et al. 2011; Kim et al. 2014; Dong et al. 2015). In 2008, a study with commercial *Echinacea* extracts revealed activities against human pathogenic bacteria (Sharma et al. 2008) and recently, a study conducted in Norwegian mothers revealed no risk of malformations or adverse pregnancy outcomes after the use of *Echinacea* in pregnancy (Heitmann et al. 2016). In relation to the chemical studies, several metabolites such as caffeic acid, chlorogenic acid and alkylamides have been described as the possible responsible for its biological activity (Kumar & Ramaiah 2011). Glycoproteins, alkylamides and polysaccharides in roots of *E. purpurea* are chemical compounds that are responsible for their immunomodulatory properties (Balciunaite et al. 2015). It is important to mention that the chemical and pharmacological analyses carried out for these plants were executed in independent studies, so the active ingredients and its pharmacological action cannot be correlated directly, hence the main objective of this work was to carry out a comparative study of three commercial extracts of *Echinacea*, quantifying the major chemical constituents in each one and, in parallel, to determine *in vivo* anti-inflammatory and hypoglycaemic effects, as well as *in vitro* antiproliferative effect.

## Materials and methods

### Biological material and experimental set up

Three commercial hydroalcoholic *Echinacea* extracts were evaluated; these extracts were obtained from certified trading houses which are subjected to trademarks. These *Echinacea* hydroalcoholic extracts will be denoted as A, B and C extracts. According to the product label summary, A was prepared with roots of *Echinacea purpurea*; B with roots, leaves, flowers and seeds of *Echinacea purpurea* and C with aerial parts and roots of *Echinacea purpurea* and roots of *Echinacea angustifolia*.

This study included physical, chemical and biological analyses. The amount of dry matter and the density of the extracts were determined for physical analysis. Qualitative and quantitative determinations were carried out in the chemical analysis. The presence of saponins, anthraquinones, alkaloids, coumarins, and tannins were determined qualitatively. The compounds determined quantitatively were: total phenolics, total flavonoids, caffeic and chlorogenic acids, sugars and alkylamides as described below. Finally, for the analysis of the biological activity the antioxidant capacity was determined by DPPH and ABTS^·+ ^methods, and the anti-inflammatory effect, the hypoglycaemic potential, and the antiproliferative effect were evaluated.

### Physical analysis of the extracts

#### Density

The density of the extracts was determined using a pycnometer equipped with a thermometer. First, the empty pycnometer was weighed, then filled with distilled water and weighed again. Finally, the pycnometer was filled with each *Echinacea* extract and weighed. Density was calculated using the following formula: *ρ*_extract_ = [W_(pycnometer + extract)_−W_pycnometer_]/[W_(pycnometer + water)_ −W_pycnometer_] [*ρ*_water_].

#### Dry matter

To determine the amount of dry matter in the extracts, 10 mL of the studied extract were dried by evaporation to constant weight in sterile conditions using a fume hood at room temperature (25 ± 2 °C). Then, the dry matter was weighed and expressed as grams of dry matter per mL of extract.

#### Identification and quantification of alcohol present in the extracts

For the determination of the type of alcohol used in the formulation of the commercial extracts, each extract was analyzed with ^1^H-NMR, obtaining the spectrum from each of the samples. The proportion of alcohol in the sample was determined by the same protocol used for the determination of sugars described below.

#### UV/Vis spectrum

A Jenway UV/Vis spectrophotometer was used to obtain the absorption spectra of the extracts. The spectrum of each extract in the wavelength range from 190 to 1100 nm was evaluated.

### Chemical analysis of the extracts

#### Qualitative analysis

##### Determination of saponins

To determine the presence of saponins in the extracts of *Echinacea*, the technique described by Coolborn and Bolatito (2010) was followed. Dried extract (0.02 g) was placed in a tube containing 10 mL of distilled water, then incubated in a water bath at 80 °C during 30 min. Afterwards, the tube was allowed to cool at room temperature, stirred vigorously and left to stand for 15 to 20 min. The presence and level of saponins were assessed by measuring the height of the foam formed.

##### Determination of anthraquinones

Determination of anthraquinones was carried out using the method of thin layer chromatography. Silica gel plates 60F_254_ of 3 × 5 cm were cut and an aliquot (0.1 mL) of each *Echinacea* extract was applied. The eluent mixture consisted of dichloromethane and methanol 95:5 (v/v). Yellow or red fluorescent spots under UV-light indicated the presence of anthraquinones (Coolborn & Bolatito 2010).

##### Determination of alkaloids

An aliquot of 0.1 mL of each *Echinacea* extract was applied on silica gel 60F_254_ plates (3 × 5 cm). Plates were eluted with the same mixture used for anthraquinones and revealed with the Dragendorff reagent. Formation of red-brown spots indicated the presence of alkaloids (Coolborn & Bolatito 2010).

##### Determination of tannins

Each extract (0.02 g) was dissolved in 10 mL of distilled water. The solution was divided into three test tubes and treated with: a gelatin solution 1% (w/v) in test tube number 1; a gelatin-salt reagent (1 g of gelatin and 10 g of NaCl dissolved in 100 mL of distilled water) in test tube number 2; saline solution [NaCl 10% (w/v)] in test tube number 3. The appearance of a white precipitate in test tubes number 1 & 2 and the absence of such precipitate in test tube number 3 indicated the presence of tannins (Coolborn & Bolatito 2010).

##### Determination of coumarins

Each extract (0.02 g) was added to 10 mL of distilled water in test tubes. These test tubes were covered with filter paper moistened in a caustic soda solution (1 g in 15 mL) and heated until boiling point. After 5 min, the filter paper was removed from the tube, dried and exposed to UV-light. Blue fluorescence indicated the presence of volatile coumarins (Coolborn & Bolatito 2010).

#### Quantitative chemical analysis

##### Total phenols by spectroscopic analysis

The content of total phenolic compounds was determined using the Folin–Ciocalteu reagent as described by Singlenton and Rossi (1965). An aliquot of 200 μL of the hydroalcoholic extracts of *Echinacea* was diluted with methanol 80% (v/v). This dilution was mixed with 1 mL of Folin–Ciocalteu reagent (previously diluted with water 1:10 (v/v)) and incubated for 1 min at room temperature, added 0.8 mL of sodium carbonate 7.5% (w/v) were added. The reaction mixture was incubated for 1 h at room temperature and subsequently the absorbance was determined at 765 nm. The standard curve was prepared with gallic acid, concentrations ranging from 0 to 200 μM. The results were expressed as mg gallic acid equivalent per mL of each extract.

##### Total flavonoids by spectroscopic analysis

Total flavonoids were determined using the colorimetric method of aluminum chloride described by Chang et al. (2002). Each hydroalcoholic *Echinacea* extract (0.5 mL) was mixed with 1.5 mL of 95% ethanol (v/v), 0.1 mL of 10% aluminum chloride (w/v), 0.1 mL of 1 M potassium acetate and 2.8 mL of distilled water. The mixture was incubated at room temperature for 30 min and the absorbance was determined at 415 nm. The standard curve was prepared with quercetin, concentrations ranging from 10 to 100 μg/mL. The results were expressed as mg quercetin equivalent per mL of each extract.

##### Determination of chlorogenic acid and caffeic acid by HPLC analysis

The determination of these two polyphenols was carried out by high performance liquid chromatography (HPLC), using the technique described by Pellati et al. (2011). The extracts were filtered through 0.45 μm nylon filters. Afterwards, these filters were analyzed microscopically. The filtrate (20 μL) was injected into an HPLC system (Agilent Technology 1260) consisting of a vacuum degasser, quaternary pump, autosampler, thermostatted column compartment and a Multiple Wavelength Detector (MWD). The chromatograms were recorded using Agilent OpenLab EZChrom 2014 Chemstation software. The analyses were carried out on waters μBondapak C18 column (10 μm, 3.9 × 300 mm) using water and acetonitrile mixture as the mobile phase in gradient elution mode. The mobile phase was composed of 0.1% (v/v) acetic acid in MilliQ water (A) and acetonitrile HPLC grade (B). The elution gradient was modified as follows: initial 15% B; from 15 to 30% B for 10 min; from 30 to 65% B for 8 min; from 65 to 80% B for 7 min; from 80 to 90% B for 5 min and 90% B isocratically for 5 min. The postrunning time was 3 min. The flow rate was 1 mL min^−1^ and the column temperature was set at 30 °C. The results were recorded at 320 nm, and they were interpolated in a standard curve of caffeic or chlorogenic acid (0.05–100 ppm). Results were expressed as ppm g^−1 ^dry weight.

##### Determination of sugars by HPLC

Each extract was filtered through 0.45 μm nylon filters (Millex, Millipore, Bedford, VA). The filtrate (20 μL) was injected into the HPLC system mentioned above. The analyses were carried out on an Agilent Hi-Plex Ca column (8% crosslinked, 7.7 × 300 mm, 8 μm) using MilliQ water as the mobile phase in an isocratic mode. The flow rate was 0.6 mL min^−1^ and the column temperature was set at 85 °C. Results were expressed as ppm sugar determined g^−1^ of dry weight. 

##### Determination of the alkylamides by GC-MS analysis

Lipophilic fraction of the hydroalcoholic *Echinacea* extracts was obtained to analyze its constituents, mainly alkylamides. Liquid–liquid extraction was carried out; 10 mL of each one of the hydroalcoholic extracts was subjected to threefold extraction with 20 ml *n-*hexane-ethyl acetate (1:1 v/v) in a separation funnel. The organic phases of each extraction were collected and concentrated in a rotatory evaporator and filtered previously to the GC analysis. GC-MS spectra were recorded on an Agilent Technologies 6890N instrument (Santa Clara, CA) consisting of an Agilent Technologies 5975B mass-selective detector using a HP5MS column (30 m × 0.25 mm ×0.25 μm) and He as carrier gas (5 μL/min). The conditions for recording spectra and identifying constituents were similar to those published by Hudaib et al. (2002). Compounds identification was performed with NIST MS Search software v 2.2, and with the EI mass spectra as reported by Bauer et al. (1988). 

### Pharmacological assays

#### Determination of antioxidant capacity by the DPPH method

The method used to test the antioxidant capacity in three replicates of each hydroalcoholic *Echinacea* extract was based on the evaluation of the free radical scavenging capacity of the extracts according to the method described by Brand-Williams et al. (1995). DDPH is a stable free radical and the assay can accommodate a large number of samples in a short period of time and it is sensitive enough to detect active principles at low concentrations. A solution of 0.1 mM DPPH (2,2-diphenyl-1-picrylhydrazyl) in methanol was prepared. An aliquot of 50 μL of trolox or hydroalcoholic extract was added to 950 μL of this solution. Diluted samples of the hydroalcoholic extract in 80% aqueous methanol (v/v) were used. The antioxidant activity was measured by decreasing the absorbance at 515 nm (Beckman DU-650, UV-Vis spectrophotometer, Brea, CA). The standard curve was prepared with trolox, concentrations ranging from 0 to 30 μM. The results were expressed as trolox equivalent antioxidant capacity (TEAC).

#### Determination of antioxidant capacity by the ABTS^·+ ^method

This method is based on the evaluation of the free radical scavenging capacity of each hydroalcoholic *Echinacea* extracts, to reduce the radical cation ABTS^·+ ^to ABTS according to the method described by Rivero-Pérez et al. (2007). The radical was generated by the reaction of 7 mM solution of ABTS in deionized water with 2.45 mM K_2_S_2_O_8_ (1:1 v/v). The solution was held in darkness at room temperature for at least 16 h to obtain stable absorbance values at 734 nm. Subsequently, PBS buffer 1 X, pH 7.4 was used to set the absorbance of this solution at 0.7 (water as blank). An aliquot of 100 μL of trolox or each hydroalcoholic extract was added to 1000 μL of this solution. Diluted samples of each hydroalcoholic extract in PBS buffer 1 X, pH 7.4 were used. The antioxidant activity was measured by the decrease in the absorbance at 734 nm (Beckman DU-650, UV-Vis spectrophotometer, USA). The standard curve was prepared with trolox, concentrations ranging from 0 to 20 μM. The results were expressed as trolox equivalent antioxidant capacity (TEAC).

#### Determination of the carrageenan-induced paw edema anti-inflammatory *in vivo* model

Inflammation was induced in 40 male Wistar rats of 84 days of age with weights between 300 and 320 g, the treatment was administered *ad libitum* during 11 days, divided into the following groups (*n* = 8): negative control group (500 mL water per group), positive control group (indomethacin 10 mg/kg in 500 mL water per group), three *Echinacea* extract groups, one for each *Echinacea* extract was analyzed (1.66 mL of the hydroalcoholic extracts in 500 mL water, this corresponds to a daily dose of 0.4 mL of extract per kilogram of each group). Inflammation was induced in the right paw by a sub-plantar injection of 0.1 mL of carrageenan 1% (w/v) in saline solution 0.9% (w/v) (Cai et al. 2014). The change in the oedema was calculated by the difference of the measured diameter between the non-swollen left paw and right paw treated with carrageenan. This study was carried out every 24 h during 11 days. The animals were provided by the animal centre at Universidad Autónoma Metropolitana Unidad-Iztapalapa. The handling of the laboratory animals was performed in agreement with the statutes of the CICUAL (Institutional Committee for the Care and Use of the Animals) based in the international and national rules established in the ‘Official Mexican Rule’ for the care and use of the laboratory animals [NOM-062-ZOO-1999] (NOM 1999).

#### Determination of the hypoglycaemic potential *in vivo* model

Diabetes was induced in 40 male Wistar rats of 84 days of age with weights between 300 and 320 g by an intraperitoneal injection of alloxan monohydrate dissolved in 0.9% (w/v) saline solution at a dose of 130 mg/kg. One week after the injection, the glucose levels in blood samples obtained from rats’ tail were monitored using a manual glucometer (ECLIPSE Infopia, Korea) to corroborate that the rats were diabetic. The diabetic rats were divided randomly into the following groups (*n* = 8) for treatment during 33 days: negative control group (500 mL water per group), positive control group (5 mg/kg glibenclamide in 500 mL water per group), three *Echinacea* groups (1.66 mL of the hydroalcoholic extracts in 500 mL water, this corresponds to a daily dose of 0.4 mL of extract per kilogram of each group). Monitoring of glucose levels was performed every 72 h during 33 days. During the study animals were fed *ad libitum* (Shah & Khan 2014). Analysis of the data was conducted by comparison of means of the groups treated with the controls at 2, 6, and 12 days of treatment.

The animals were provided by the animal centre at Universidad Autónoma Metropolitana Unidad-Iztapalapa. The handling of the laboratory animals was performed in agreement with the statutes of the CICUAL (Institutional Committee for the Care and Use of the Animals) based on the international and national rules established in the ‘Official Mexican Rule’ for the care and use of the laboratory animals [NOM-062-ZOO-1999] (NOM 1999).

#### Antiproliferative effect in tumor cell lines

MCF-7, HeLa and HCT-15 cells were maintained in RMPI 1640 medium with 10% (w/v) fetal bovine serum and cultured at 37 °C in an atmosphere of 5% CO_2_ in air (100% humidity). Cells at log phase of growth cycle were treated in triplicate with five different concentrations of the biological dry material adjusted in DMSO (dry material was obtained by evaporating 1 mL of each *Echinacea* extract using a nitrogen flow), and incubated for 72 h at 37 °C in a 100% humidified atmosphere and 5% CO_2_. Cell growth was determined by the sulforhodamine B assay (SRB) in 96-well plates. Sulforhodamine B absorbance was measured at 564 nm. This colorimetric method expresses the percentage of proliferation in cells treated with the *Echinacea* extracts compared with a DMSO blank, using the following formula, % cell growth = (A_t_-A_b_)/(A_c_-A_b_) × 100, where A_t _=_ _absorbance value of treatment or positive control (colchicine) well, A_b _=_ _absorbance value of DMSO blank well, A_c _=_ _absorbance value of growth control well (Skehan et al. 1990; Mendoza-Espinoza et al. 2009; Campos-Lara & Mendoza-Espinoza 2011).

### Statistical analysis

Statistical analysis was carried out using NCSS software (2007, update 2009, v07.1.18). Data were compared applying one-way ANOVA followed by Tukey’s multiple comparison tests. Values were considered as statistically significant at *p* < 0.05.

## Results and discussion

Given that this study was performed with commercial *Echinacea* hydroalcoholic extracts, the presence of ethyl alcohol was determined by ^1^H-NMR and the proportion of this alcohol in the preparation was determined by HPLC. 0.45 μm nylon filters were analyzed microscopically and no abnormal particles were found. Table 2 shows the percentage of ethyl alcohol detected in each extract. Qualitative and quantitative chemical analysis indicated that extract C prepared with *E. purpurea* and *E. angustifolia* is richer in phenols and total flavonoids (Table 2), this result correlates with the higher antioxidant activity found in the *in vitro* analyses of this extract (Table 2). The correlation between the content of phenols and total flavonoids with the antioxidant capacity has also been proposed by Kumar et al. (2008) and Farasat et al. (2014) in ten Indian medicinal plants and edible green seaweeds, respectively.

**Table 2.** Evaluation of physical and chemical parameters and biological activity of *Echinacea* extracts.

	Biological material^a^		
	A	B	C
Physical			
Dry matter (g/mL)	0.024	0.027	0.239
Density (g/mL)	0.933	0.930	0.978
Ethyl alcohol^b^	40%	40%	30%
Chemical			
Qualitative*^*c*^*			
*Saponins*	+	++	+
*Coumarins*	+	–	+
*Anthraquinones*	–	+	+
*Tannins*	+	–	+
Quantitative			
*Total phenols*^d^	23.3 ± 0.1	17.7 ± 0.1	137.5 ± 2.3
*Total flavonoids*^e^	0.16 ± 0.01	0.16 ± 0.003	0.62 ± 0.02
*Caffeic acid*^f^	–	–	–
*Chlorogenic acid*^f^	–	<1.0	<1.0
*Alkylamides*^g^	11	11	14
*Sugars*^f^			
Sucrose	267	77.95	68061.22
Glucose	1660	2475.07	2521.65
Fructose	6788.36	3554.74	29938.56
Biological			
Antioxidant capacity^h^			
DPPH	0.24 ± 0.01	0.45 ± 0.02	4.8 ± 0.4
ABTS^+^	1.66 ± 0.06	1.26 ± 0.04	10.5 ± 0.2
Cytotoxicity^i^			
HeLa	>30,000	>30,000	270.8
MCF-7	>30,000	>30,000	8,171
HCT-15	>30,000	>30,000	9,338

^a^Extract produced in a certified ranch using: A, roots of *Echinacea purpurea*; B, roots, leaves, flowers and seeds of *Echinacea purpurea*; C, aerial part and roots of *Echinacea purpurea* and roots of *Echinacea angustifolia*. ^b^The alcohol type was determined by ^1^H-RMN and the percentage was determined by HPLC. ^c^Qualitative analysis: +++ very abundant; ++ abundant; + scarce; - not oserved. Quantitative analysis: ^d^mEAG/mL, equivalent mg of gallic acid per milliliter of extract. ^e^mEQ/mL, equivalent mg of quercetin per milliliter of extract. ^f^PPM determined by HPLC as indicated in the experimental section. ^g^Number of alkylamides found by GC-MS. ^h^Trolox Equivalent Antioxidant Capacity, equivalent millimolar per milliliter of extract. ^i^Half inhibitory concentration (IC_50_) in μg/mL.

**Figure 1.**
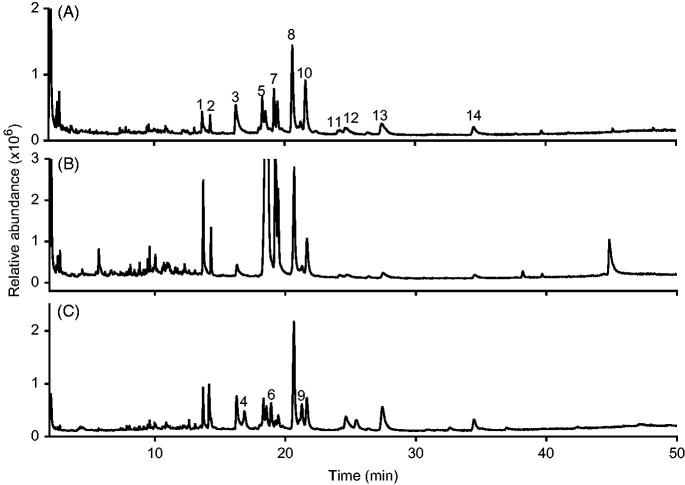
Total ion chromatogram (TIC) of the non-polar fractions of the analyzed hydroethanolic extracts: roots of *E. purpurea* (A), roots and aerial parts of *E. purpurea* (B) and roots, aerial parts of *E. purpurea* and roots of *E. angustifolia* (C). The identity of the detected peaks is shown in Table 3.

Gas chromatography analysis coupled to mass spectrometry (GC-MS) revealed the presence of at least 11 alkylamides in extracts A and B and 14 alkylamides in extract C (Figure 1). The concentration and type of alkylamides depends on the *Echinacea* species, as well as the plant tissue. A previous study performed by Pellati et al. (2011) using a similar extraction method in whole *E. purpurea* plants, reported 22 alkylamides in *E. angustifolia* and 17 in *E. purpurea*. Mudge et al. (2011) have reported that roots of *E. angustifolia* have a broader variety of alkylamides compared to the roots of *E. purpurea* which coincides with our results showing the detection of 11 alkylamides in extract A which is only constituted by *E. purpurea* roots, while in extract C (constituted of a mixture of roots of *E. purpurea* and *E. angustifolia*) 14 alkylamides were detected from which three are exclusive of *E. angustfolia* (Figure 1 and Table 3).

**Table 3.** Identity, retention times and presence of alkylamides on the non-polar fractions of three *Echinacea* extracts analyzed GC-MS.

Peak	Rt (min)	Compound	A	B	C
1	13.69	undeca-2*E*,4*Z*-diene-8,10-diynoic acid isobutylamide	X	X	X
2	14.12	undeca-2*E*-ene-8,10-diynoic acid isobutylamide	X	X	X
3	16.25	dodeca-2*Z*,4*E*-diene-8,10-diynoic acid isobutylamide	X	X	X
4	16.87	undeca-2Z-ene-8,10-diynoic acid isobutylamide			X
5	18.09	undeca-2E,4Z-diene-8,10-diynoic acid 2-methylbutylamide	X	X	X
6	18.89	dodeca-2*E*,4*E*-dienoic acid isobutylamide			X
7	19.46	dodeca-2*E*-ene-8,10-diynoic acid isobutylamide	X	X	X
8	20.65	dodeca-2*E*,4*E*,8*Z*,10*Z*-tetraenoic acid isobutylamide	X	X	X
9	21.25	dodeca-2*E*,4*Z*,8*Z*,10*E*-tetraenoic acid isobutylamide			X
10	21.60	dodeca-2*E*,4*Z*,8*Z*,10*Z*-tetraenoic acid isobutylamide^a^	X	X	X
11	24.66	undeca-2*Z*,4*E*-diene-8,10-diynoic acid 2-methylbutylamide^a^	X	X	X
12	25.46	dodeca-2*E*-ene-8,10-diynoic acid 2- methylbutylamide	X	X	X
13	27.467	dodeca-2*E*,4*Z*-diene-8,10-diynoic acid isobutylamide	X	X	X
14	34.44	dodeca-2*E*,4*Z*-diene-8,10-diynioc acid 2-methylbutylamide	X	X	X

a The identity of this compounds cannot be determined with a good grade of accuracy, there is a fair probability these compounds are isomers of other compounds of the same molecular weight and similar fragmentation pattern.

In the model of paw edema in male Wistar rats, extract B (elaborated from roots, leaves, flowers and seeds of *Echinacea purpurea*) showed a robust anti-inflammatory effect compared to the negative control on day 2 and 3; however, it was less powerful than the positive control (indomethacin) (see support material). It is important to note that the effect of this extract was observed until day 2 and 3 of the treatment, which may indicate that *Echinacea* extracts have an anti-inflammatory effect but slower than the indomethacin positive control. The other two extracts A and C, showed no significant differences compared to the negative control, which could indicate that the active metabolite for this activity is possibly located in the leaves of *Echinacea purpurea* and not in the root as it has been reported in some ethnobotanical studies of *Echinacea* (World Health Organization 1999). These results agree and support the study of the protective effect of *Echinacea* against hepatotoxicity caused by diethylnitrosamine, reported by Rezaie et al. (2013) and the protective effect on induced colitis reported by Dogan et al. (2014). The effect observed by these research groups can be attributed to the anti-inflammatory effect found on the present work; however, it has not been specifically determined in an *in vivo* anti-inflammatory study. The role of the alkylamides on this effect is not clear, since extract C (with the highest content of alkylamides) showed no anti-inflammatory effect. On the other hand, the anti-inflammatory activity may be due to the abundant presence of saponins observed in the qualitative analysis of extract B. Saponins are a group of heterogeneous secondary metabolites of plants and consist of triterpenoid or steroid aglycone moiety and complex oligosaccharide substituents. Among other properties, the saponins have been associated with anti-inflammatory activity in some Nigerian medicinal plants and in blue cohosh (Hassan et al. 2012; Lee et al. 2012). It is worth mentioning that on day 1, indomethacin had a significantly higher anti-inflammatory effect than the three evaluated extracts. Generally, this effect could be attributed to the fact that drugs contain higher concentrations of active principles compared with the content in herbal extracts.

A hypoglycaemic effect was observed for the three hydroalcoholic extracts, being extract A the most promising. The average glycemia of male Wistar rats treated with extract A was lower than the positive control (glibenclamide), while rats treated with extracts B and C showed similar average values between them and a similar tendency as the positive control along the experimental period (Table 4).

**Table 4.** Hypoglycaemic effect of the three extracts evaluated.

	Measurement day		
	2	6	12
A	116.5 ± 6.9^a,b^	124.8 ± 9.5^a,b^	133.25 ± 10.04^a,b^
B	112.8 ± 19.3^a,b^	295.0 ± 43.2	317.5 ± 21.5
C	125.0 ± 54.4^a,b^	232.5 ± 131.6^a,b^	232 ± 135.4^a,b^
Control	412.3 ± 11.1	387.0 ± 72.4	436.25 ± 58.78
Glibenclamide	140.0 ± 34. 6^a^	104.0 ± 15.2^a^	147.75 ± 31. 91^a^

Glycaemia average (mg/dL) ± SD, ANOVA, Tukey as *post hoc* test, *n* = 8, ^a^Significant difference compared to control (=0.005, *p* = 1.000, see Supporting information); ^b^No difference compared to glibenclamide (=0.05, *p* = 1.000).

In relation to the antiproliferative effect, dry material was obtained by evaporating 1 mL of each extract using a nitrogen flow. IC_50_ (half inhibitory concentration) of A and B were above 30,000 μg/mL, while C showed values in the range from 500 to 10,000 μg/mL, being much more toxic for HeLa cell line and moderately toxic for HCT15 and MCF-7 cell lines. Antiproliferative effect of C may be attributable to the higher number of alkylamides detected by GC-MS analysis (Table 3). It is important to mention that these results correlate with the ability to inhibit the growth of human pancreatic cancer cell lines reported by Chicca et al. (2007). The different antiproliferative effect observed in the extracts could be explained by the difference in the type and concentration of alkylamides. Usually, this kind of compounds is related with this biological effect.

**Figure 2.**
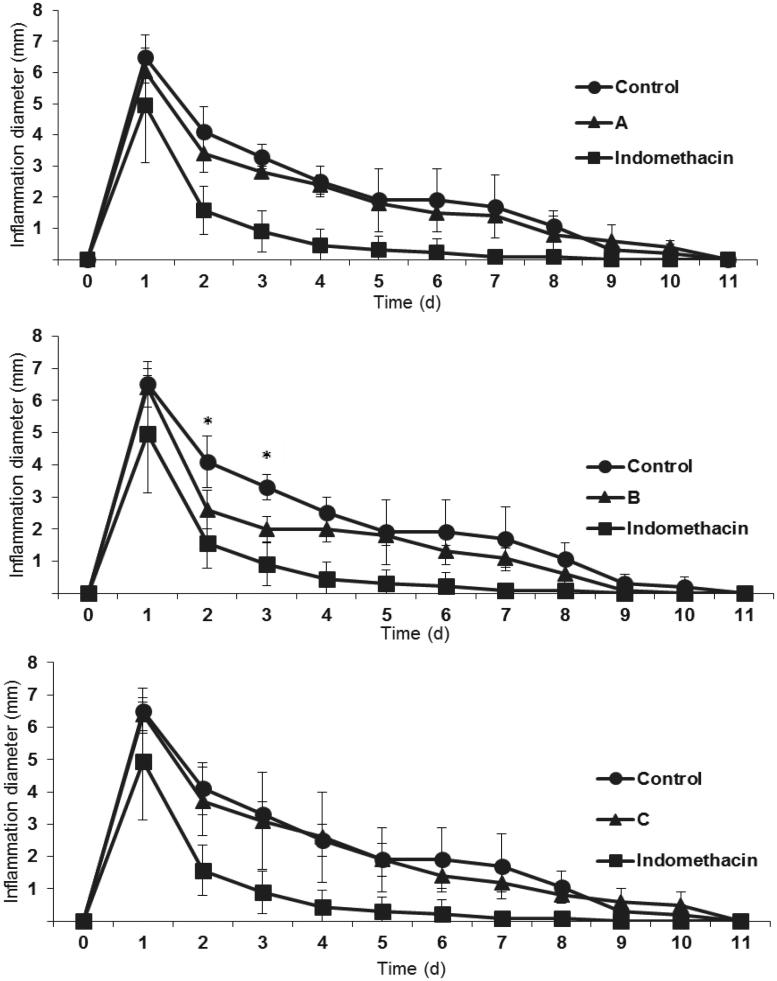
Anti-inflammatory effect of *Echinacea* extracts (A–C) in the paw oedema model. *indicates significant differences at *α* = 0.05, *n* = 8.

UV spectra of the extracts were obtained (Figure 2) in order to have their chemical fingerprints. There is a high similarity among the spectral lines of the three analyzed extracts; therefore, the composition of the biological material present in the extracts could not be determined by this method.

## Conclusion

There are differences in the physical, phytochemical and biological properties of the three evaluated commercial *Echinacea* extracts. In this study, 11 alkylamides were identified in extracts A and B and 14 alkylamides in extract C (Table 3 and Figure 1).

Only extract B showed an anti-inflammatory effect, which suggest that the active compound for this effect is probably present in the aerial part of *E. purpurea*. Even though extract C was also prepared with aerial parts of *E. purpurea*, no anti-inflammatory effect was observed. Probably, this is an effect of dilution of extract C which was prepared with two species of *Echinacea* (aerial parts and roots of *E. purpurea* and roots of *E. angustifolia*) and possibly masks the anti-inflammatory effect observed in extract B.

The hypoglycaemic effect found in the extracts A and C could be attributed to the roots of *E. purpurea*. The present work represents the first report where *Echinacea* is associated with this pharmacological effect. It would be interesting to perform specific studies to determine the bioactive compounds and to elucidate the mechanism of action of this effect.

A moderate antiproliferative effect was only observed in extract C; the effect correlates with the highest content of alkylamides found in the non-polar fraction of this hydroalcoholic extracts. The results presented here evidence the need to consider strategies and policies of standardization, not only aimed to guarantee the safety and identity of the botanical products, but also their pharmacological effect. It is important to note that the three evaluated extracts of *Echinacea* are recommended in a similar way; however, its chemical content and biological effects are very different as shown in the present work.
